# Prognostic Value of Epidermal Growth Factor Receptor Mutations in Resected Non-Small Cell Lung Cancer: A Systematic Review with Meta-Analysis

**DOI:** 10.1371/journal.pone.0106053

**Published:** 2014-08-27

**Authors:** Zhixuan Zhang, Ting Wang, Jun Zhang, Xiaohong Cai, Changchuan Pan, Yu Long, Jing Chen, Chengya Zhou, Xude Yin

**Affiliations:** 1 Department of Medical Oncology, Sichuan Cancer Hospital & Institute, Chengdu, Sichuan, PR China; 2 Department of Radiation Oncology, Sichuan Cancer Hospital & Institute, Chengdu, Sichuan, PR China; H. Lee Moffitt Cancer Center & Research Institute, United States of America

## Abstract

**Background:**

The prognostic value of epidermal growth factor receptor (EGFR) mutations in resected non-small cell lung cancer (NSCLC) remains controversial. We performed a systematic review with meta-analysis to assess its role.

**Methods:**

Studies were identified via an electronic search on PubMed, Embase and Cochrane Library databases. Pooled hazard ratio (HR) for disease-free survival (DFS) and overall survival (OS) were calculated for meta-analysis.

**Results:**

There were 16 evaluated studies (n = 3337) in the meta-analysis. The combined HR evaluating EGFR mutations on disease free survival was 0.96 (95% CI [0.79–1.16] *P* = 0.65). The combined HR evaluating EGFR mutations on overall survival was 0.86 (95% CI [0.72–1.04] *P* = 0.12). The subgroup analysis based on univariate and multivariate analyses in DFS and OS showed no statistically significant difference. There was also no statistically significant difference in DFS and OS of stage I NSCLC patients.

**Conclusion:**

The systematic review with meta-analysis showed that EGFR mutations were not a prognostic factor in patients with surgically resected non-small cell lung cancer. Well designed prospective study is needed to confirm the result.

## Introduction

Lung cancer is a major public health problem all over the world. In 2014, there were an estimated 224,210 new cases with lung and bronchus cancer in the United States, in addition, the estimated deaths from lung cancer were 159,260 [1]. On top of that, more than 80% of lung cancer cases are of non-small cell lung cancer (NSCLC) [2] and it was estimated 51% of patients present with advanced disease at the time of diagnosis [3]. Although much progress had been made in optimizing the treatment of NSCLC (including multidisciplinary therapy, targeted therapy and etc), the 5-year overall survival rate remained about 15% of all stages [1]. Therefore it is important to search for new therapies that will improve the current overall treatment in battling against NSCLC.

In recent years, a few biomarkers have emerged as prognostic or predictive factors in non-small cell cancer which include epidermal growth factor receptor (EGFR), ALK (Anaplastic Large Cell Lymphoma) fusion Gene, K-ras oncogene and others. However, a meta-analysis demonstrated that Ras gene alteration is a poor prognostic factor for survival in NSCLC [4]. On the other hand, among these biomarkers, EGFR gene mutations have been the center of the majority researches in assessing its role as a prognostic or predictive factor in NSCLC. As we know, EGFR gene mutations are a predictive factor for epidermal growth factor tyrosine kinase inhibitor (EGFR-TKI) therapy in advanced NSCLC, which was confirmed by the IPASS trial [5]. Still, it is unclear whether EGFR mutations are a prognostic factor in earlier-stage NSCLC patients who underwent surgical resection. In order to clarify the prognostic value of EGFR mutation status for survival, we performed the systematic review of the literature with methodological assessment and meta-analysis.

## Materials and Methods

### Search strategy and selection criteria

Our research used the PRISMA (Preferred Reporting Items for Systematic Reviews and Meta-Analyses) statement as a guide. In order to be eligible for the review, study should evaluate the relationship of EGFR mutation status and patient survival in resected NSCLC. Articles were identified via an electronic search on PubMed, Embase and Cochrane Library. Two investigators (ZX Zhang, XH Cai) performed the search independently. The search started from the articles incepted and ended on March 2014. We used the following keywords: “EGFR or epidermal growth factor receptor or HER1 or erB1” and “NSCLC or lung cancer or lung carcinoma or lung neoplasm” and “resected” in our search. We also performed manual search for the articles in the reference. We only searched the studies that were published in English. Studies included in the meta-analysis had to meet the following criteria: 1) All patients had pathologically proven localized NSCLC with stage I-III; 2) All patients received complete resection; 3) EFGR gene mutations are detected in all patients; 4) hazard ratios (HR) for disease-free survival (DFS) and overall survival (OS) could be found in articles or could be calculated by related parameters. Patients were excluded if they had received tyrosine kinase inhibitors (TKIs) as neo-adjuvant treatment or adjuvant treatment. Abstracts and unpublished studies were excluded. If the author reported results which obtained on the same patient population in several studies, we would use the most recent or complete study.

### Quality Assessment

For biological prognostic factors for lung cancer, we used the European Lung Cancer Working Party (ELCWP) quality scale, which was used by Steels et al [6], to assess the trial methodology. The scale had four main categories: scientific design, laboratory methodology, generalizability and results analysis. Each category had a few items. Except when specified, the attributed value per item was 2 points if it was clearly stated in the article, 1 point if its description was incomplete or unclear, if it was not defined or inadequate defined then it would be 0 point. Each category had a maximum score of 10 points; the overall maximum theoretical score was 40 points. If an item was not applicable to a study, its value could not be taken into account for the category. The final scores were expresses as percentage, the higher value of the article indicated a better methodological quality. The quality assessment was performed by two investigators (ZX Zhang, J Zhang) independently.

### Data Extraction

Data extraction used a standard form by two investigators (ZX Zhang, T Wang) independently. Any discrepancies were solved via discussion. When a study could be included in the meta-analysis, a consensus must be reached by both investigators. The main characteristics extracted from articles were: first author, year of publication, source of patients, patients number, histological type, pathologic stage, median follow-up time (months), rate of patients with EGFR mutations, EGFR mutation status, detecting methods, hazard ratio estimation and survival result. If author reporting univariate and multivariate analysis results for survival, we would use the latter ones. As a few factors as pathological stage, age, performance status were known as prognostic factor [7]; the multivariate analysis would eliminate the effect of other prognostic factors on survival. If the author gave the result of survival analysis with or without patients received TKI treatment for tumor recurrence, we would use the latter to reduce the effect.

### Statistical Method

The study was considered significant when the *P*-value for the statistical test comparing survival distributions between the groups with and without EGFR mutations was <0.05. The survival result of the study would be defined as “positive” when EGFR mutations were a favorable prognostic factor for survival. On the contrary, when EGFR mutations were a poor prognostic factor for survival, it would be defined as “negative”. *P*-value≥0.05 meant EGFR mutations were not a prognostic factor for survival in which it was termed “not significant”.

We used nonparametric tests to compare the distribution of the quality scores according to the value of a discrete variable (binary variables were calculated by Mann-Whitney tests). The primary end point was DFS and the secondary end point was OS. DFS was defined as periods calculate from the date of surgery until the date of recurrence and death or the last follow-up. We used combined HRs to measure the impact of EGFR mutations on DFS and OS. For each study, HR and 95% confidence interval (CI) were estimated from the publications. Some studies supplied the HR and 95% CI directly, while other studies they were acquired by calculating the following parameters: the total number of events, the number of patients at risk in each group and the log-rank statistic or its *P*-value. Then we calculate the log (HR), SE (log (HR)), Variance, O-E statistic (difference between numbers of observes and expected events) according to the methods described by Tierney [8]. If the only exploitable data was survival curves, we would analyze it by using Enguage Digitizer version 4.1. We used the Cochran Q statistic (a *P*-value<0.10 called significant for heterogeneity) and I^2^ value to assess heterogeneity among the studies. I^2^>50% was considered significant heterogeneity. Fixed-effect model was used firstly for calculating pooled HR, if the assumption of homogeneity had to be rejected, a random-effect model would be used. A pooled HR<1 implied a better survival for the group with EGFR mutations. If the 95% CI for overall HR overlapped 1 was considered not significant. All statistical analysis was performed by Review manager 5.0 (http://www.cochrane.org). Sensitivity analysis was done to explore the influence of each study to survival outcomes. Subgroup analysis was performed to explore the influence of statistical analysis method and pathologic stage in the outcomes. We only performed analysis for stage I for there was no studies for stage II and stage III. We used the funnel plot and Begg’s test to assess the publication bias by Stata 11.0. If authors only reported the results in subgroups, the article was treated as a separate study.

## Results

### Selection and characteristics of studies

**Figure 1:**
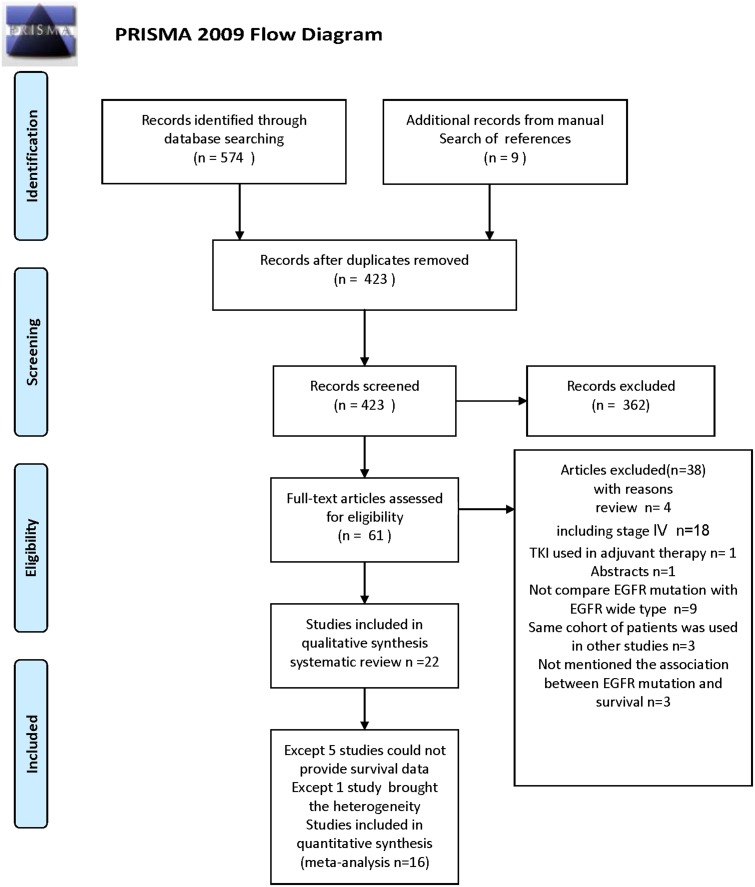
PRISMA Flow chart of the search result of the meta-analysis.

A total of 583 potentially relevant articles were searched from electronic database and 9 from manual search of reference. After reading the title and abstract, 61 articles were selected to read the full articles by two investigators (ZX Zhang, XD Yin), 3 of them were excluded as the cohort was similar in other articles (studies excluded [9], [10], [11]; studies included [12], [13], [14]. Finally, 22 studies were found eligible for the systematic review which were published between 2007 and 2014 [15], [13], [16]–[24], [12], [25]–[32], [14], [33], in which 5 studies could not provide sufficient data for meta-analysis [16], [19], [20], [32], [14]. Figure 1 shows the flow chart of the search result.

The main characteristics and results of the eligible studies are summarized in table 1. The total number of patients was 4122, ranging from 53 to 733. The rate of EGFR mutations was 3.4%–59.5%. EGFR mutations were found more frequently in Asians than in Caucasians (41.7% and 13.98% respectively), though there were only 9 Asians included in study [26]. The rates of EGFR mutations were 26.19% in NSCLC and 40.76% in adenocarcinoma (ADC). 5 studies were stage I, one study was stage IIIA, and others were stage I–III. 12 studies reported HR and 95% confidence interval (CI). We needed extract the data from survival curves to reconstruct the HR estimate and its variance in 4 studies. We calculated the HR and log-rank *P*-value from one study. 3 studies concluded that EGFR gene mutation status was a positive prognostic factor. 19 studies reported EGFR gene mutations were not a prognostic factor for survival. For detecting mutations of EGFR gene, PCR and direct sequencing were used.

**Table 1:** Characteristics of the eligible studies.

First author	Year	Source of patients	Patients number	Histology	Pathologic stage	Median follow-up time(Months)	Rate of EGFR mutations (%)	EGFR mutation status	Detecting methods	Hazard ratio estimation	Survival result	
											DFS	OS
Suehisa	2007	japan	187	ADC	I–IIIA	56.2 control group; 65.7 adjuvant chemotherapy group	42.2	19,21	PCR SEQ	HR	No data	NS
Na II	2007	korea	133	NSCLC	I–III	29	24	18–21	PCR SEQ	Survival curves	No data	NS
Sonobe	2007	japan	53	NSCLC	I–IIIA	More than 60	32	18–21	PCR SSCP	No data	No data	NS
Marks	2008	USA	244	ADC	I–III	35	14	18–21	PCR ddSEQ Sequenom	HR	No data	NS
Kobayashi	2008	japan	127	ADC	I	No daa	50.4	19,21	EPCR PAGE	HR	NS	NS
Sasaki	2009	japan	109	NSCLC	I-III	No data	50.5	No data	PCR SEQ	No data	No data	NS
Galleges	2009	Netherlands	148	NSCLC	I–III	No data	3.4	18–21	Nested- PCR SEQ	No data	No data	NS
Hosokawa	2009	japan	93	NSCLC	I–III	No data	40	18–21	PCR SEQ	Survival curves	No data	NS
Lee	2009	korea	117	ADC	I–IIIA	40.3	45.3	18–21	Nested-PCR NSEQ	HR	Positive	NS
D’Angelo	2010	USA	733	ADC	I-III	16	14.5	19,21	PCR SEQ	HR	No data	NS
Liu	2010	Taiwan	164	NSCLC	I–IIIA	27 days to 158 months	31.7	18–21	PCR SEQ	HR	No data	NS
Koh	2010	korea	130	NSCLC	I-III	113	19.2	18,19,21	PCR SEQ	HR	NS	NS
Tsao	2011	Canada	221	NSCLC	IB–II	No data	12.2	19,21	PCR SEQ SLA ARMS	HR	NS	NS
Izar	2013	USA	307	NSCLC	I	30	20.2	18–21	SEQ Multiplex PCR	HR	Positive	Positive
Sun	2013	China	150	NSCLC	IIIA	No data	28.7	19,21	RT-PCR	HR	Positive	Positive
Ragusa	2013	Italy	102	ADC/BAC	I–III	37	19.6	18–21	Nested-PCR SEQ	HR	NS	NS
Dong	2013	China	301	ADC	IA-IIIA	79.1	52.5	18,19,21	RT-PCR SEQ	Survival curves	NS	NS
Maki	2013	Japan	105	ADC	IA	59.7	48.6	No data	MNE-PCR	HR+*P*	NS	NS
Ohba	2014	Japan	242	ADC	I	No data	47.7	19,21	PCR SEQ	Survival curves	NS	NS
Yang	2014	Taiwan	163	ADC	I	At least 40	59.5	18–21	PCR SEQ	No data	No data	NS
Kim	2014	Korea	162	ADC	IB-IIIA	No data	50.6	18–21	Nested-PCR	No data	NS	NS
Liu	2014	China	131	ADC	I-IIIA	40.9	44.3	18–21	Nested-PCR	HR	NS	NS

PCR: polymerase chain reaction; Sequenom: mass spectrometry-based genotyping; ddSEQ: direct dideoxynucleotide sequencing; EPCR: mutation-enriched PCR; PAGE: polyacrylamide gel electrophoresis; NSEQ: nucleotide sequencing; SLA: sensitivity fagment length analysis; ARMS: amplified refractory mutation system; MNE-PCR: mutant non-enriched PCR; NS: nonsignificant.

### Quality Assessment

The results of the methodological assessment according to the ELCWP score are shown in table 2. A total of 22 studies were eligible for systematic review. 17 studies that provided sufficient data were eligible for meta-analysis. The overall global score ranged from 42.5% to 65% with a median of 56%. The global Score between 17 evaluated studies and 5 studies that were not evaluated for meta-analysis was not statistically significant difference (*P* = 0.38). No statistically significant difference was shown between the Asian and Non-Asian studies according to the global score (54.5% and 60% respectively, *P* = 0.089). There was no significant difference between the nonsignificant and significant studies (median global score 55.53% versus 59.17%, *P* = 0.411). We can perform a quantitative aggregation of the survival results because of the absence of a significant quality difference between significant and nonsignificant studies.

**Table 2:** Results of the methodological assessment by the European Lung Cancer Working Party score.

	Studiesnumber	GlobalScore (%)	Design*	Laboratorymethodology*	Generalizability*	Resultsanalysis*
All studies	22	56.0	5.27	5.36	6.54	5.13
Evaluated for meta-analysis	17	56.6	5.35	5.35	6.64	5.29
Not evaluated for meta-analysis	5	54.0	5.0	5.4	6.2	4.6
*P*-value		0.38	0.35	0.87	0.33	0.05
Asian	16	54.5	5.25	5.18	6.44	5.06
Non-Asian	6	60.0	5.33	5.83	6.83	5.33
*P*-value		0.089	0.74	0.20	0.64	0.49
Nonsignificant	19	55.53	5.32	5.32	6.42	5.05
Significant	3	59.17	5.0	5.67	7.33	5.66
*P-*value		0.411	0.550	0.651	0.236	0.215

Scores in the table are summarized by the median values. *: scored out of 10. Significant: the *P*-value for the statistical test comparing survival distributions between the groups with and without EGFR mutation was<0.05. Not significant: the *P*-value≥0.05 meant EGFR mutation was not a prognostic factor for survival.

### Disease free survival

**Figure 2:**
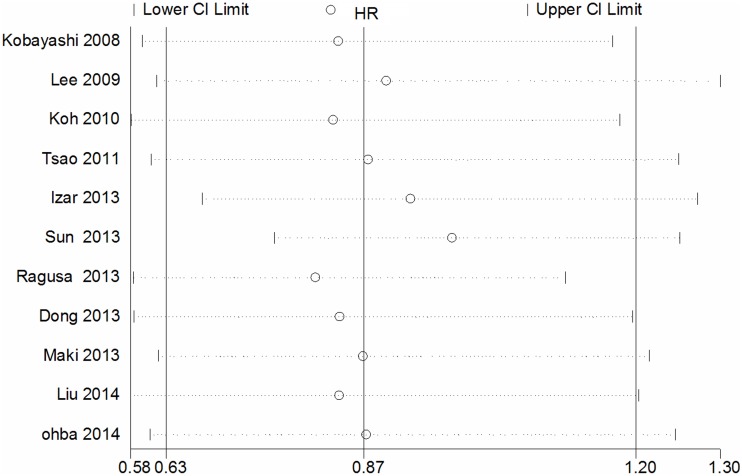
Sensitivity analysis for combined HRs evaluating EGFR mutations on disease free survival.

**Figure 3:**
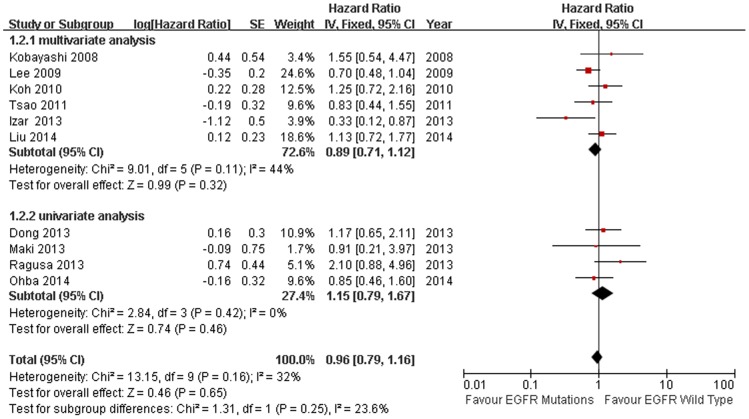
Fixed-effect model forest plot of HR of DFS in statistical analysis method subgroup analysis according to EGFR mutation status. Solid diamond indicates the pooled HR of DFS, square indicates hazard ratio value of each study.

**Figure 4:**
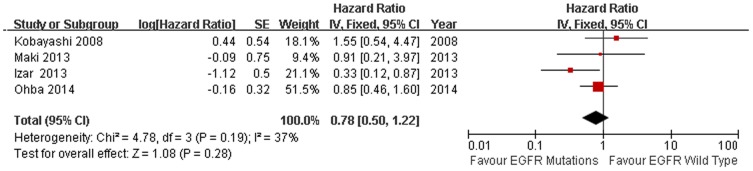
Fixed-effect model forest plot of HR of DFS in pathologic stage subgroup analysis according to EGFR mutation status. Solid diamond indicates the pooled HR of DFS, square indicates hazard ratio value of each study.

**Figure 5:**
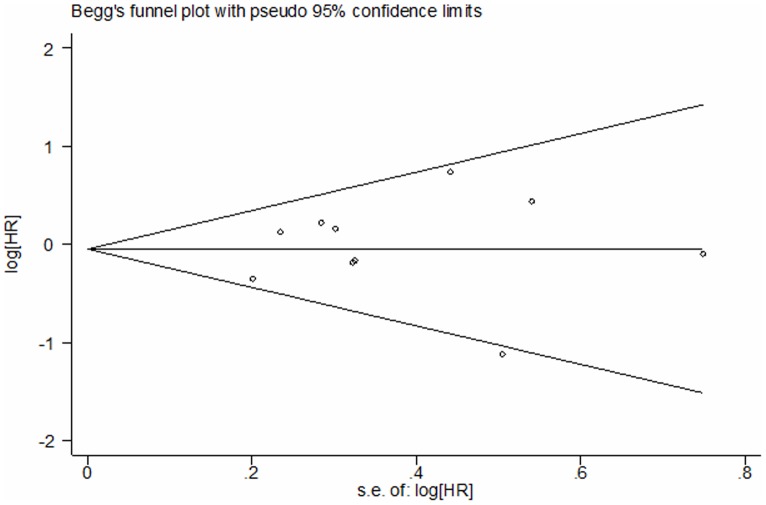
Funnel plot for publication bias test DFS. The two oblique lines indicate the pseudo 95% CI.

11 studies compared the DFS between EGFR mutations and wild-type groups [18], [22], [12], [25], [26], [27], [28], [29], [30], [31], [33]. Significant heterogeneity was detected among the studies (I^2^ = 62%, *P* = 0.003). We explored the source of heterogeneity by using sensitivity analysis [Figure 2]. One study that investigated EGFR mutations in stage IIIA NSCLC solely was the main source of heterogeneity [27]. The value of I^2^ ranged from 59% to 66% if the study was included. After excluding this study, there was no significant heterogeneity among the studies (I^2^ = 32%, *P* = 0.16). The remaining 10 studies were used to perform meta-analysis. The HR was calculated by using fixed-effect model. The combined HR evaluating EGFR mutations on DFS was 0.96 (95% CI [0.79–1.16] *P* = 0.65, Figure 3), indicating that EGFR mutations were not a prognostic factor for DFS. The subgroup analysis was performed according to the statistical analysis method in the survival outcomes and pathologic stage. Fixed-effect model was used in subgroup analysis. Multivariate analysis was used by 6 studies [18], [22], [12], [25], [26], [33], and univariate analysis was used in 4 studies [28], [29], [30], [31]. There was no significant association between EGFR mutations and DFS (multivariate analysis HR = 0.89, 95% CI [0.71–1.12] *P* = 0.32; univariate analysis HR = 1.15, 95% CI [0.79–1.67] *P* = 0.46 Figure 3). In the subgroup analysis according to pathologic stage, there was no significant heterogeneity among the 4 studies (I^2^ = 37%, *P* = 0.19) [18], [26], [30], [31]. There was no association between EGFR mutations and DFS in stage I NSCLC patients. (HR = 0.78, 95% CI [0.50–1.22] *P* = 0.28 Figure 4). No significant publication bias were observed in the funnel plots and Begg’s test (*P* = 0.72, Figure 5).

### Overall Survival

**Figure 6:**
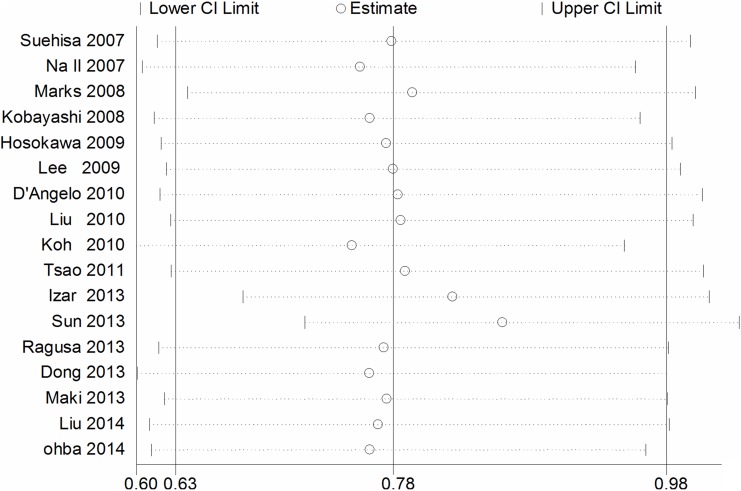
Sensitivity analysis for combined HRs evaluating EGFR mutations on overall survival.

**Figure 7:**
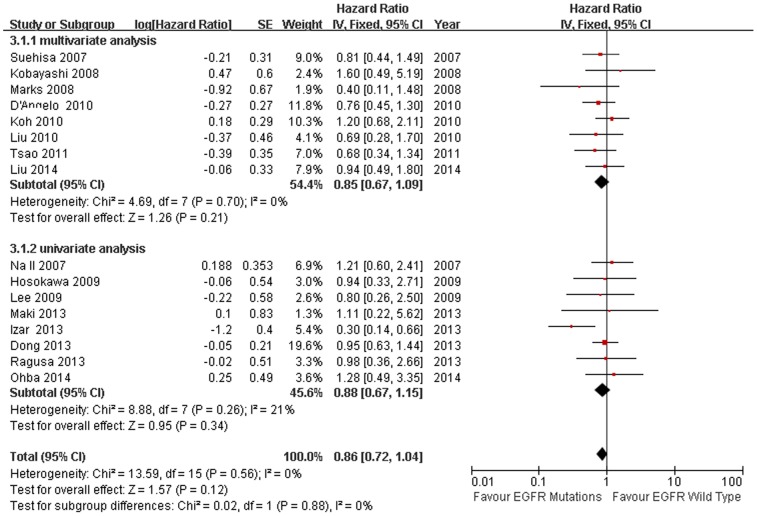
Fixed-effect model forest plot of HR of OS in statistical analysis method subgroup analysis according to EGFR mutation status. Solid diamond indicates the pooled HR of OS, Square indicates hazard ratio value of each study.

**Figure 8:**
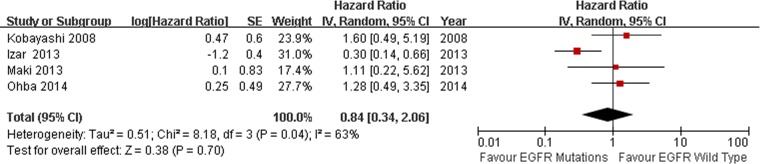
Random-effect model forest plot of HR of OS in pathologic stage subgroup analysis according to EGFR mutation status. Solid diamond indicates the pooled HR of OS, Square indicates hazard ratio value of each study.

**Figure 9:**
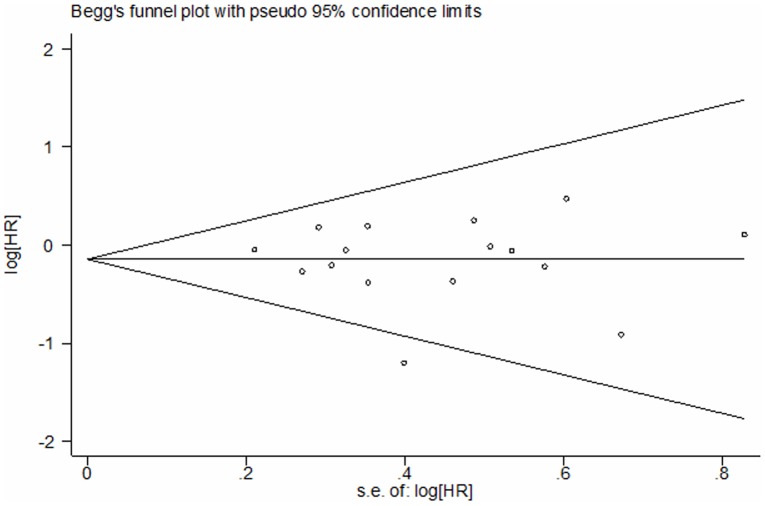
Funnel plot for publication bias test OS. The two oblique lines indicate the pseudo 95% CI.

All the studies compared the median survival time. We conducted sensitivity analysis (Figure 6), however, there was no significant heterogeneity was found in the studies (I^2^ = 35%, *P* = 0.08). We found there was no heterogeneity among the studies when one study was excluded [27] (I^2^ = 0%, *P* = 0.56). The combined HR and 95% CI did not alter when excluding any study but one [27]. We thought the different pathologic stage maybe the main reason. After excluding the Sun study, the remaining 16 studies were used to perform the meta-analysis. We used the fixed-effect model to calculate the HR. The combined HR evaluating EGFR mutations on overall survival was 0.86 (95% CI [0.72–1.04] *P* = 0.12; Figure 7). The subgroup analysis was also performed according to the statistical analysis method in the survival outcomes and pathologic stage. Multivariate analysis was used by 8 studies, while univariate analysis was used in 8 studies. There was no significant heterogeneity in each subgroup, I^2^ was 0% and 21% respectively. Fixed-effect model was used in subgroup analysis. The result of multivariate analysis did not show association between EGFR mutations and OS (HR = 0.85, 95% CI [0.67–1.09] *P* = 0.21, Figure 7); the result of univariate analysis also did not show the association (HR = 0.88, 95% CI [0.67–1.15] *P* = 0.34, Figure 7). 4 studies were stage I NSCLC, significant heterogeneity was found in the subgoup (I^2^ = 63%, *P* = 0.04). We used the random-effect model in the subgroup analysis. There was no association between EGFR mutations and OS in stage I NSCLC subgroup. (HR = 0.84, 95% CI [0.34–2.06] *P* = 0.70 Figure 8). No significant publication bias were observed in the funnel plots and Begg’s test (*P* = 0.739, Figure 9).

## Discussion

Surgical approach is the only reasonable method in treating the patients with earlier stage of NSCLC. Platinum-based adjuvant chemotherapy is the standard treatment but it only has a 5-year absolute benefit of 5.4% compared with surgery alone [34]. In 2004, EGFR mutations were found to have a predictive effect for the treatment of EGFR-tyrosine kinase inhibitor (EGFR-TKI) in advanced NSCLC [35], [36]. Some studies demonstrated that EGFR mutations seemed to have a favorable prognostic value on survival in advanced NSCLC [37], [38]. It is interesting to discuss the acting role of EGFR mutation in stage I-III NSCLC, whether it serves as a predictive factor or prognostic factor. If it has a prognostic value, it would enable us to better identify patients’ risk of recurrence, furthermore, guiding us to make an optimal and individualized treatment plan for the patients. That being said, the prognostic value of EGFR mutations remains controversial in resected NSCLC patients [18], [24], [26], therefore it is essential in gathering more data in order to analysis and expound its role as a prognostic factor. Our result demonstrated that EGFR mutations were not a prognostic factor in resected NSCLC patients.

It is the first meta-analysis conducted on the prognostic value of EFGR mutations in resected stage I-III NSCLC. There were 16 evaluated studies (n = 3337) which were included in the meta-analysis. The article may serve as a reference for the adjuvant therapy of EGFR-TKI. When a therapy is initiated, not only the predictive role of a marker in clinical outcome should be studied, but also its prognostic role should be considered [39]. Overall survival is considered to be the most reliable indicator to evaluate survival outcome but it requires long-term follow-up. All the studies that were included in the meta-analysis were retrospectively conducted, not only the follow-up time was different, the patient follow-up time was relatively short as well in some of the studies. The heterogeneity of treatment for recurrent disease would cause an impact on OS, especial the usage of EGFR-TKI in our study. We thought it was appropriate to use DFS rather than OS as the primary end point of the study. We chose patients who did not receive EGFR-TKI therapy pre- or postoperatively. The pooled hazard ratio of EGFR mutations on DFS was not affected by using EGFR-TKI. We performed the meta-analysis after the methodological assessment by ELCWP scale which could avoid some selection biases. The scale was designed for biological prognostic factors which based on the experts’ opinions and years of experience in the field. We could perform a quantitative aggregation of the survival results as there was no significant difference between significant and nonsignificant studies. 5 studies were excluded for meta-analysis due to insufficient data to estimate the HR which may bring about the publication bias. However, all of the 5 excluded studies reported EGFR mutations were not the prognostic factor. Thus, the result of the meta-analysis was not affected.

The significant heterogeneity was found in the 11 studies eligible for meta-analysis of DFS. We thought two factors contributed to the heterogeneity. The sensitivity analysis showed one study maybe one of the causes of heterogeneity [27]. It is necessary to explore the real prognostic value of EGFR mutation status in patients with surgically resected stage I NSCLC. There were only five studies included patients with stage I, four of them demonstrated EGFR mutation status was not prognostic factor [18], [30], [31], [32], one in the four studies had insufficient data to calculate the HR [32]. Only one study showed EGFR mutation status was a positive prognostic factor [26]. Data was scarce and we were unable to combine the datum to perform meta-analysis on patients with stage I. Therefore we included patients with stage I-III NSCLC. Most of the studies included pathologic stage I and II except for one study which it only involved stage IIIA NSCLC [27]. We believed there was difference in the selection of patients. At the same time, there were nearly half of the patients enrolled in the study did not receive chemotherapy after resection of stage IIIA-N2 NSCLC tumor which it would affect the survival outcomes. Other important factors were the frequency of follow-up and the imaging methods. The time interval for surveillance was ranged from 3 months to 6 months postoperatively. The follow-up time was associated with the discovery time of disease recurrence: the shorter the follow-up time was, the earlier we could detect disease recurrence. In addition, some of the studies used chest x-ray and abdomen ultrasound as the imaging workup while others used thoracic computed tomography scan. Using cranial computed tomography/magnetic resonance imaging also affected the discovery time of disease recurrence. We couldn’t analyze the subgroups of both frequency of follow-up and imaging methods, as many studies did not give an account of sufficient detailed information.

Many factors would influence the result of the meta-analysis such as the baseline characteristics of the patients (including age, sex, pathologic stage, smoking history, pathological subtype). Smoking is the major co-founding factor for overall survival for lung cancer patient. 8 studies included 1937 patients on OS had multivariate analysis and two of them which included 977 patients were only analyzed after stage controlled [17], [23]. Thus the result of meta-analysis has to be treated cautiously. It is critical to comprise more studies to update and consummate the data in the future.

The techniques used to detect EGFR mutations may bring the bias. In NSCLC, common methodologies used to detect the EGFR mutations are: direct sequencing, PCR-SSCP, mutant-enriched PCR, ARMS, microfluidics digital PCR, HRM, DHPLC, and etc. There are pros and cons in each method; not to mention the rate of mutation is different in each method as well [40]. Some research, for the purpose of improving the detection rate, applied several methods [25]. There was difference in detecting mutations within exons of the EGFR gene. Many evidences showed that patients with advanced lung adenocarcinomas harbor EGFR mutations, L858R missense mutations at exon 21 or deletions in exon 19 were more sensitive to the EGFR-TKI erlotinib or gefitinib [36], [40], [41]. Most studies in the meta-analysis examined exons 18–21. There were a few studies and some subgroup analysis in articles gave the result of exon 19 deletions or L858R EGFR sensitive mutations [15], [18], [23], [24], [25], [28], [31], [33]. Most of them showed no relationship between EGFR sensitive mutations and survival outcomes. One study showed it had borderline significance by multivariate analysis (*P = *0.0506) [24]. Another study demonstrated in the ADC/BAC group, EGFR sensitive mutations showed tendency towards a worse disease-free survival (*P*  = 0.056) [28]. The subgroup analysis of one study showed the EGFR mutations did not have significant prognostic value but patients with exon 19 mutation tended to have better prognosis compared to patients with exon 21 mutation ((*P = *0.056) [33].

There were other biases found in the meta-analysis. Only the full published papers were included. Unpublished papers and meeting abstracts were excluded for insufficient data. The method of extrapolation of HR may be another potential source of bias. One study reported the HR and log-rank *P* value; we could utilize the given value and calculate the survival rates. 4 studies supported the survival curves alone, and it was imprecise to extract survival rates from it.

In conclusion, the systematic review with meta-analysis suggested that EGFR mutations were not a prognostic factor in surgically resected NSCLC patients. Well designed prospective study is needed to confirm the result. We could evaluate the prognostic value of EGFR mutation in a more homogenous population of patients with postoperative stage I disease (especially stage IA) for eliminating the interference of any other treatment factors.

## Supporting Information

Checklist S1 (DOC)
