# Supplementary material for: Prognostic Value of Epidermal Growth Factor Receptor Mutations in Resected Non-Small Cell Lung Cancer: A Systematic Review with Meta-Analysis
Source: PLoS One. 2014 Aug 27;9(8):e106053. doi: 10.1371/journal.pone.0106053 (PMC4146589; doi:10.1371/journal.pone.0106053)
Supplement: Checklist S1 — (DOC) [file pone.0106053.s001.doc]

| **Section/topic** | **#** | **Checklist item** | **Reported on page #** |
| --- | --- | --- | --- |
| **TITLE** | | |  |
| Title | 1 | Prognostic Value of Epidermal Growth Factor Receptor Mutations in Resected Non-Small Cell Lung Cancer: A Systematic Review with Meta-Analysis | Title |
| **ABSTRACT** | | |  |
| Structured summary | 2 | **Background:** The prognostic value of epidermal growth factor receptor (EGFR) mutations in resected non-small cell lung cancer (NSCLC) remains controversial. We performed a systematic review with meta-analysis to assess the role of it. **Methods:** Studies were identified via an electronic search on PubMed, Embase and Cochrane Library databases. Pooled hazard ratio (HR) for disease-free survival (DFS) and overall survival (OS) were calculated for meta-analysis. ***Results:*** There were 16 evaluated studies (n=3337) in the meta-analysis. The combined HR evaluating EGFR mutations on disease free survival was 0.96 (95%CI [0.79-1.16] *P*=0.65). The combined HR evaluating EGFR mutations on overall survival was 0.86 (95%CI [0.72-1.04] *P*=0.12). The subgroup analysis based on univariate and multivariate analyses in DFS and OS showed no statistically significant difference. There was also no statistically significant difference in DFS and OS of stage I NSCLC patients. | Abstract |
| **INTRODUCTION** | | |  |
| Rationale | 3 | Lung cancer is a major public health problem all over the world. In recent years, a few biomarkers have emerged as prognostic or predictive factors in non-small cell cancer, including epidermal growth factor receptor (EGFR), ALK (Anaplastic Large Cell Lymphoma) fusion Gene, K-ras oncogene and others. Among these biomarkers, most researches are about EGFR gene mutations. As we all known, EGFR gene mutations are a predictive factor for epidermal growth factor tyrosine kinase inhibitor (EGFR-TKI) therapy in advanced NSCLC, which was confirmed by the IPASS trail. But it is unclear whether EGFR mutations are a prognostict factor in earlier-stage, who undergo surgical resection. | Introduction |
| Objectives | 4 | In order to clarify the prognostic value of EGFR mutation status for survival, we performed the systematic review of the literature with methodological assessment and meta-analysis. | Introduction |
| **METHODS** | | |  |
| Protocol and registration | 5 | This research use the PRISMA (Preferred Reporting Items for Systematic Reviews and Meta-Analyses) statement as a guide.(available at：http://www.prisma-statement.org) | Materials and Methods |
| Eligibility criteria | 6 | Cohort studies and RCTs, published in English only. | Materials and Methods |
| Information sources | 7 | Articles were identified via an electronic search on PubMed, Embase and Cochrane Library. The search started from the articles incepted and ended on March 2014. | Materials and Methods |
| Search | 8 | We used the following keywords: “EGFR or epidermal growth factor receptor or HER1 or erB1” and “NSCLC or lung cancer or lung carcinoma or lung neoplasm” and “resected”. We also used manual search for the articles in the reference. We only searched the articles published in English. | Materials and Methods |
| Study selection | 9 | Studies included in the meta-analysis had to meet the following criteria: 1) All patients had pathologically proven localized NSCLC with stage**Ⅰ-Ⅲ** 2) All patients received complete resection 3) All patients detected EFGR gene mutations. 4) hazard ratios (HRs) for disease-free survival (DFS) and overall survival (OS) could be found in articles or could be calculated by related parameters. Patients were excluded if they had received tyrosine kinase inhibitors (TKIs) as neo-adjuvant treatment or adjuvant treatment. Abstracts and unpublished studies were excluded. If author reported results obtained on the same patients population in several studies, we used the most recent or complete study. | Materials and Methods |
| Data collection process | 10 | Data extraction was conducted independently by 2 investigators and Quality of the studies was assessed using by European Lung Cancer Working Party quality scale for biological prognostic factors for lung cancer (ELCWP) by 2 independent evaluators. Data were adjudicated by 2 additional investigators according to the original articles after data extraction and assessment. Any disagreement will be present to discuss within all authors. | Materials and Methods |
| Data items | 11 | First author, year of publication, source of patients, number of patients, histological type, pathologic stage, median follow-up time (months), number of patients with EGFR mutations, EGFR mutation status, test method, hazard ratio estimation, survival result. If original hazard ratio was not reported, the Kaplan–Meier curves would be used to extract hazard ratio estimation according to the methods described by Tierney in 2007. | Materials and Methods |
| Risk of bias in individual studies | 12 | The report quality of studies was assessed using the European Lung Cancer Working Party ((ELCWP) quality scale for biological prognostic factors for lung cancer. The quality assessment was performed by two investigators. The scale had four main categories: scientific design; laboratory methodology; generalizability; results analysis. Each categories had a few items. Except when specified ,the attributed value per item is 2 points if it is clearly in the article,1point if its description is incomplete or unclear, if it is not defined or is inadequate was 0 point. Each categories had a maximal score of 10 points, the overall maximum theoretical score was 40 points. If a item was not applicable to a study, its value could not be taken into account in the total for the category. The final scores were expresses as percentages, the higher values of the article means a better methodological quality. | Materials and Methods |
| Summary measures | 13 | Hazard Ratio | Materials and Methods |
| Synthesis of results | 14 | The association between EFGR mutations and PFS or OS was expressed as a hazard ratio (HR).  Statistical heterogeneity between studies was examined using both the Cochrane Q statistic (significant at *P*<0.1) and the I^2^ value. I^2^ >50% were considered to represent significant heterogeneity respectively. A fixed-effect model was used when heterogeneity was not detected by chi-square test (*P* >0.10); otherwise, a random-effect model was used. All statistical analysis was performed by Review manager 5.0 (http://www.cochrane.org). The pooled OR and its 95% confidence intervals (CIs) were calculated using Mantel–Haenszel formula (fixed-effect model) or Dersimonian–Laird formula (random-effect model). HR was used to measure the impact of EGFR mutations on DFS or OS. HR, its variance, its 95% CI, log (HR) and se(log(HR)) for each study were extracted or calculated based on the published researches according to the methods described by Tierney. Kaplan-Meier curves were read by Engauge Digitizer version 4.1 (http://digitizer.sourceforge.net/). A significant two-way *P* value for comparison was defined as *P*<0.05. The results were described by forest plots, every square represents each study’s HR estimate. The pooled HR is symbolized by a solid diamond at the bottom of the forest plot and the width of the square represents the 95% CI of HR. The size of the square represents the weight that the corresponding study exerts in the meta-analysis. | Materials and Methods |

Page 1 of 2

| **Section/topic** | **#** | **Checklist item** | **Reported on page #** |
| --- | --- | --- | --- |
| Risk of bias across studies | 15 | The potential publication bias was evaluated by funnel plots and Begg’s test by Stata 11.0. | Materials and Methods |
| Additional analyses | 16 | Subgroup analysis was performed to explore the influence of statistical analysis method and pathologic stage i in the outcomes**.** Sensitivity analysis was done to explore the influence of each study to survival outcomes. | Materials and Methods |
| **RESULTS** | | |  |
| Study selection | 17 | 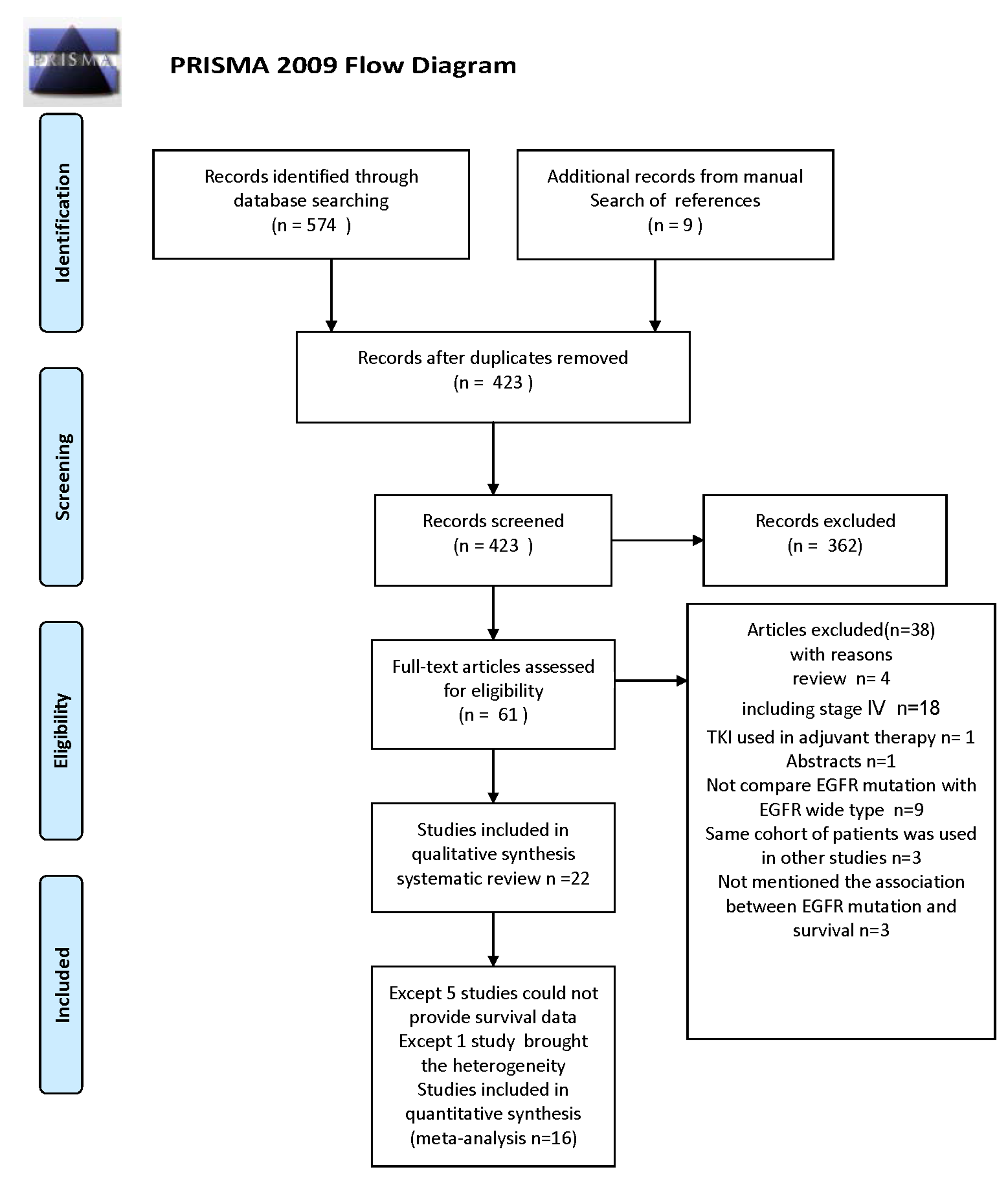 | Results |
| Study characteristics | 18 | | **First author** | **Year** | **Source of patients** | **Patients number** | **Histology** | **Pathologic stage** | **Median follow-up time(Months)** | **Rate of EGFR mutations (%)** | **EGFR mutation status** | **Detecting methods** | **Hazard ratio estimation** | **Survival result** | | | --- | --- | --- | --- | --- | --- | --- | --- | --- | --- | --- | --- | --- | | **DFS** | **OS** | | Suehisa | 2007 | japan | 187 | ADC | I-IIIA | 56.2 control group; 65.7 adjuvant chemotherapy group | 42.2 | 19,21 | PCR SEQ | HR | No data | NS | | Na Ⅱ | 2007 | korea | 133 | NSCLC | I-III | 29 | 24 | 18-21 | PCR SEQ | Survival curves | No data | NS | | Sonobe | 2007 | japan | 53 | NSCLC | I-IIIA | More than 60 | 32 | 18-21 | PCR SSCP | No data | No data | NS | | Marks | 2008 | USA | 244 | ADC | I-III | 35 | 14 | 18-21 | PCR ddSEQ Sequenom | HR | No data | NS | | Kobayashi | 2008 | japan | 127 | ADC | I | No daa | 50.4 | 19,21 | EPCR PAGE | HR | NS | NS | | Sasaki | 2009 | japan | 109 | NSCLC | I-III | No data | 50.5 | No data | PCR SEQ | No data | No data | NS | | Galleges | 2009 | Netherlands | 148 | NSCLC | I-III | No data | 3.4 | 18-21 | Nested- PCR SEQ | No data | No data | NS | | Hosokawa | 2009 | japan | 93 | NSCLC | I-III | No data | 40 | 18-21 | PCR SEQ | Survival curves | No data | NS | | Lee | 2009 | korea | 117 | ADC | I-IIIA | 40.3 | 45.3 | 18-21 | Nested-PCR NSEQ | HR | Positive | NS | | D'Angelo | 2010 | USA | 733 | ADC | I-III | 16 | 14.5 | 19,21 | PCR SEQ | HR | No data | NS | | Liu | 2010 | Taiwan | 164 | NSCLC | I-IIIA | 27 days to 158months | 31.7 | 18-21 | PCR SEQ | HR | No data | NS | | Koh | 2010 | korea | 130 | NSCLC | I-III | 113 | 19.2 | 18,19,21 | PCR SEQ | HR | NS | NS | | Tsao | 2011 | Canada | 221 | NSCLC | IB-II | No data | 12.2 | 19,21 | PCR SEQ SLA ARMS | HR | NS | NS | | Izar | 2013 | USA | 307 | NSCLC | I | 30 | 20.2 | 18-21 | SEQ Multiplex PCR | HR | Positive | Positive | | Sun | 2013 | China | 150 | NSCLC | IIIA | No data | 28.7 | 19,21 | RT-PCR | HR | Positive | Positive | | Ragusa | 2013 | Italy | 102 | ADC/BAC | I-III | 37 | 19.6 | 18-21 | Nested-PCR SEQ | HR | NS | NS | | Dong | 2013 | China | 301 | ADC | IA-IIIA | 79.1 | 52.5 | 18,19,21 | RT-PCR SEQ | Survival curves | NS | NS | | Maki | 2013 | Japan | 105 | ADC | IA | 59.7 | 48.6 | No data | MNE-PCR | HR+*P* | NS | NS | | Ohba | 2014 | Japan | 242 | ADC | I | No data | 47.7 | 19,21 | PCR SEQ | Survival curves | NS | NS | | Yang | 2014 | Taiwan | 163 | ADC | I | At least 40 | 59.5 | 18-21 | PCR SEQ | No data | No data | NS | | Kim | 2014 | Korea | 162 | ADC | IB-IIIA | No data | 50.6 | 18-21 | Nested-PCR | No data | NS | NS | | Liu | 2014 | China | 131 | ADC | I-IIIA | 40.9 | 44.3 | 18-21 | Nested-PCR | HR | NS | NS |   PCR: polymerase chain reaction; Sequenom: mass spectrometry-based genotyping; ddSEQ: direct dideoxynucleotide sequencing; EPCR: mutation-enriched PCR; PAGE: polyacrylamide gel electrophoresis; NSEQ: nucleotide sequencing; SLA: sensitivity fagment length analysis; ARMS: amplified refractory mutation system; MNE-PCR: mutant non-enriched PCR; NS: nonsignificant.   1. Suehisa H, Toyooka S, Hotta K, Uchida A, Soh J, et al. (2007) Epidermal growth factor receptor mutation status and adjuvant chemotherapy with uracil-tegafur for adenocarcinoma of the lung. [J Clin Oncol](http://www.ncbi.nlm.nih.gov/pubmed?term=(Suehisa%5BAuthor - First%5D) AND 25%5BVolume%5D) 25: 3952-3957. 2. Na II, Rho JK, Choi YJ, Kim CH, Koh JS, et al. (2007) Clinical features reflect exon sites of EGFR mutations in patients with resected non-small-cell lung cancer. J Korean Med Sci 22: 393-399. 3. Sonobe M, Nakagawa M, Takenaka K, Katakura H, Adachi M, et al. (2007) Influence of epidermal growth factor receptor (EGFR) gene mutations on the expression of EGFR, phosphoryl-Akt, and phosphoryl-MAPK, and on the prognosis of patients with non-small cell lung cancer. J Surg Oncol 95: 63-69. 4. Marks JL, Broderick S, Zhou Q, Chitale D, Li AR, et al. (2008) Prognostic and therapeutic implications of EGFR and KRAS mutations in resected lung adenocarcinoma. J Thorac Oncol 3: 111-116. 5. Kobayashi N, Toyooka S, Ichimura K, Soh J, Yamamoto H, et al. (2008) Non-BAC Component but not Epidermal Growth Factor Receptor Gene Mutation is Associated with Poor Outcomes in Small Adenocarcinoma of the Lung. J Thorac Oncol 3: 704–710. 6. Sasaki H, Shimizu S, Okuda K, Kawano O, Yukiue H, et al. (2009) Epidermal growth factor receptor gene amplification in surgical resected Japanese lung cancer. Lung Cancer 64: 295-300. 7. Galleges Ruiz MI, Floor K, Steinberg SM, Grünberg K, Thunnissen FB, et al. (2009) Combined assessment of EGFR pathway-related molecular markers and prognosis of NSCLC patients. Br J Cancer 100: 145-152. 8. Hosokawa S, Toyooka S, Fujiwara Y, Tokumo M, Soh J, et al. (2009) Comprehensive analysis of EGFR signaling pathways in Japanese patients with non-small cell lung cancer. Lung Cancer 66: 107-113. 9. Lee YJ, Park IK, Park MS, Choi HJ, Cho BC, et al. (2009) Activating mutations within the EGFR kinase domain: a molecular predictor of disease-free survival in resected pulmonary adenocarcinoma. J Cancer Res Clin Oncol 135: 1647-1654. 10. D’Angelo SP, Janjigian YY, Kris MG, Pao W, Riely GJ, et al. (2010) Impact of EGFR and KRAS mutations on survival in 1,000 patients with resected lung adenocarcinoma. J Clin Oncol 28(Suppl): 7011. 11. Liu HP, Isaac Wu HD, Chang JW, Wu YC, Yang HY, et al. (2010) Prognostic Implications of Epidermal Growth Factor Receptor and KRAS Gene Mutations and Epidermal Growth Factor Receptor Gene Copy Numbers in Patients with Surgically Resectable Non-small Cell Lung Cancer in Taiwan. J Thorac Oncol 5: 1175–1184. 12. Koh Y, Jang B, Han SW, Kim TM, Oh DY,et al. (2010) Expression of class III beta-tubulin correlates with unfavorable survival outcome in patients with resected non-small cell lung cancer. J Thorac Oncol 5: 320-325. 13. Tsao MS, Sakurada A, Ding K, Aviel-Ronen S, Ludkovski O, et al. (2011) Prognostic and Predictive value of EGFR Tyrosine Kinase Domain Mutation Status and Gene Copy Number for Adjuvant Chemotherapy in Non-Small Cell Lung Cancer. J Thorac Oncol 6: 139–147. 14. Izar B, Sequist L, Lee M, Muzikansky A, Heist R, et al. (2013) The impact of EGFR mutation status on outcomes in patients with resected stage I non-small cell lung cancers. Ann Thorac Surg 96: 962-968. 15. Sun HB, Ou W, Li Y, Fang Q, Qin J, et al. (2013) Epidermal growth factor receptor mutation status and adjuvant chemotherapy in resected advanced non-small-cell lung cancer. Clin Lung Cancer 14: 376-382. 16. Ragusa M, Vannucci J, Ludovini V, Bianconi F, Treggiari S, et al. (2013) Impact of Epidermal Growth Factor Receptor and KRAS Mutations on Clinical Outcome in Resected Non-Small Cell Lung Cancer Patients. Am J Clin Oncol: In press. 17. Dong Y, Li Y, Peng H, Jin B, Huang A, et al. (2013) Predictive role of EGFR mutation status on postoperative prognosis in patients with resected lung adenocarcinomas. Zhongguo Fei Ai Za Zhi 16: 177-183. 18. Maki Y, Soh J, Ichimura K, Shien K, Furukawa M, et al. (2013) Impact of GLUT1 and Ki-67 expression on early‑stage lung adenocarcinoma diagnosed according to a new international multidisciplinary classification. Oncol Rep 29: 133-140. 19. Ohba T, Toyokawa G, Kometani T, Nosaki K, Hirai F, et al. (2014) The mutations of the EGFR and K-ras genes in resected stage I lung adenocarcinoma and their clinical significance. Surg Today 44: 478-486. 20. Yang CY, Lin MW, Chang YL, Wu CT, Yang PC, et al. (2014) Programmed cell death-ligand 1 expression in surgically resected stage I pulmonary adenocarcinoma and its correlation with driver mutations and clinical outcomes. Eur J Cancer: In press. 21. Kim MH, Shim HS, Kang DR, Jung JY, Lee CY, et al. (2014) Clinical and prognostic implications of ALK and ROS1 rearrangements in never-smokers with surgically resected lung adenocarcinoma. Lung Cancer 83: 389-395. 22. Liu WS, Zhao LJ, Pang QS, Yuan ZY, Li B, et al. (2014) Prognostic value of epidermal growth factor receptor mutations in resected lung adenocarcinomas. Med Oncol 31: 771. | Results |
| Risk of bias within studies | 19 | |  | Studies number | Global Score (%) | Design* | Laboratory methodology* | Generalizability* | Results analysis* | | --- | --- | --- | --- | --- | --- | --- | | All studies | 22 | 56.0 | 5.27 | 5.36 | 6.54 | 5.13 | | Evaluated for meta-analysis | 17 | 56.6 | 5.35 | 5.35 | 6.64 | 5.29 | | Not evaluated for meta-analysis | 5 | 54.0 | 5.0 | 5.4 | 6.2 | 4.6 | | *P*-value |  | 0.38 | 0.35 | 0.87 | 0.33 | 0.05 | | Asian | 16 | 54.5 | 5.25 | 5.18 | 6.44 | 5.06 | | Non-Asian | 6 | 60.0 | 5.33 | 5.83 | 6.83 | 5.33 | | *P*-value |  | 0.089 | 0.74 | 0.20 | 0.64 | 0.49 | | Nonsignificant | 19 | 55.53 | 5.32 | 5.32 | 6.42 | 5.05 | | Significant | 3 | 59.17 | 5.0 | 5.67 | 7.33 | 5.66 | | *P-*value |  | 0.411 | 0.550 | 0.651 | 0.236 | 0.215 |   Scores in the table are summarized by the median values. *: scored out of 10. Significant: the *p*-value for the statistical test comparing survival distributions between the groups with and without EGFR mutation was <0.05. Not significant: the *p*-value≥0.05 meant EGFR mutation was not a prognostic factor for survival. | Results |
| Results of individual studies | 20 | 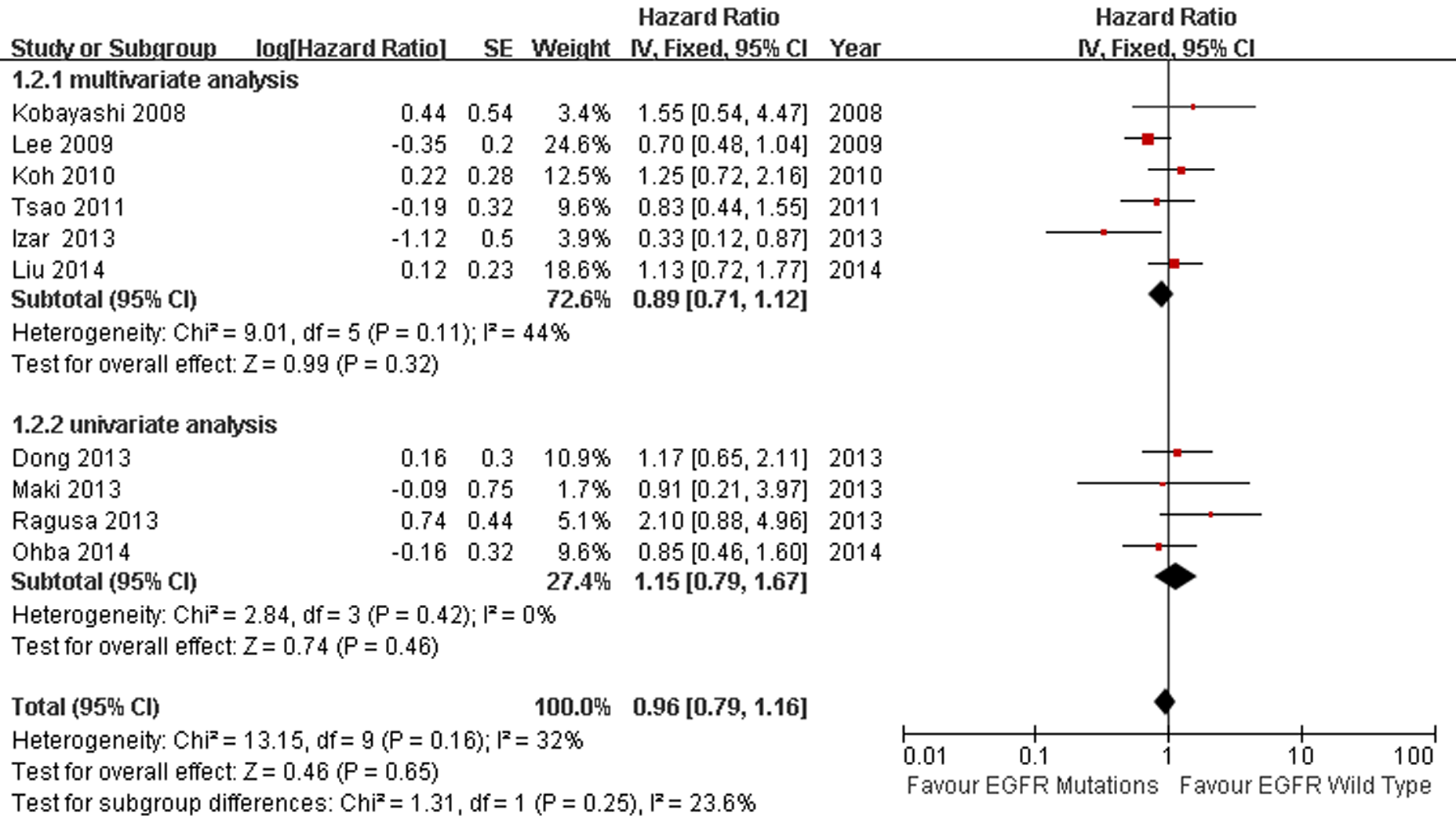  **Figure 3.** **Fixed-effect model forest plot of HR of DFS in** **statistical analysis method subgroup analysis according to EGFR mutation status**. Solid diamond indicates the pooled HR of DFS, square indicates hazard ratio value of each study.  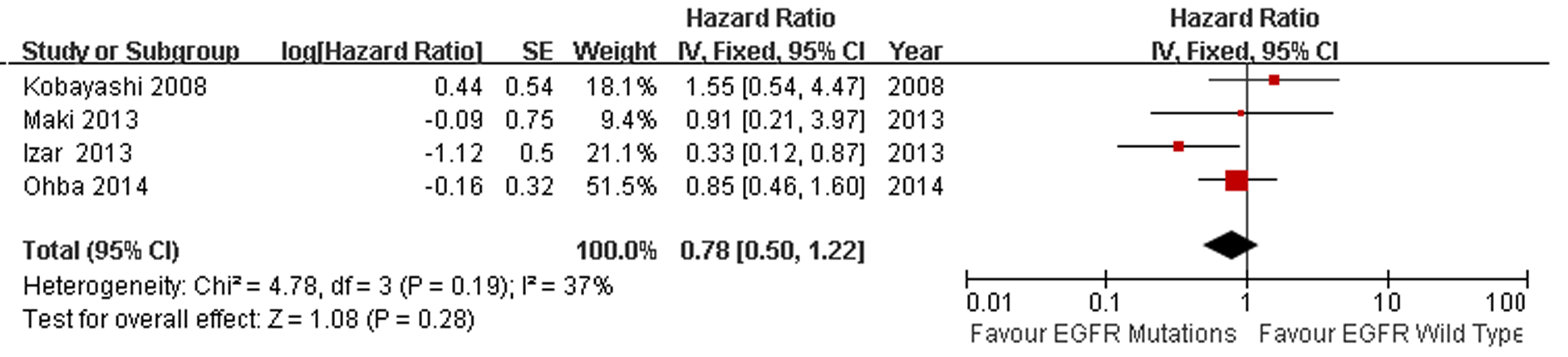  **Figure 4. Fixed-effect model forest plot of HR of DFS in pathologic stage subgroup analysis according to EGFR mutation status.** Solid diamond indicates the pooled HR of DFS, square indicates hazard ratio value of each study.  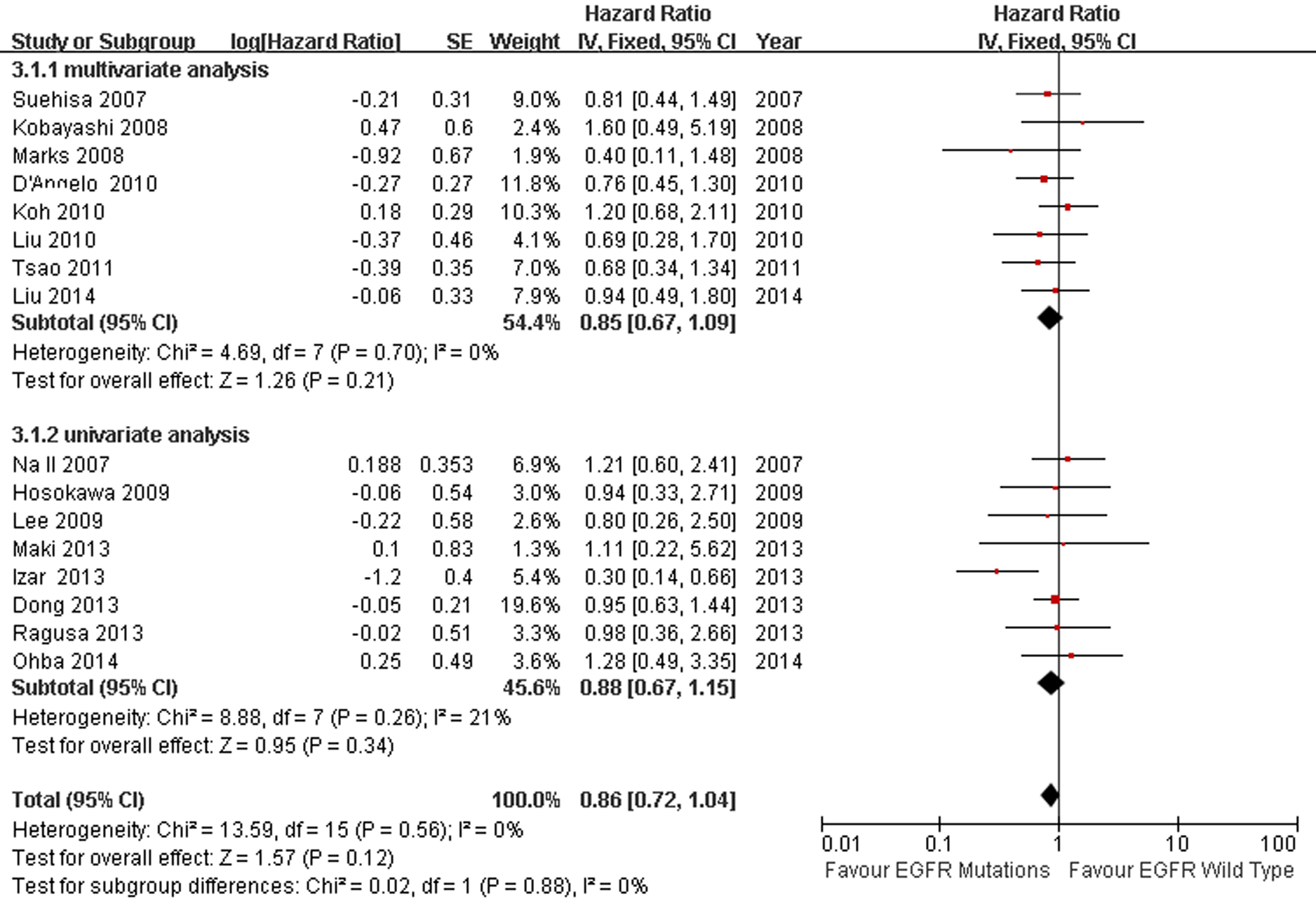  **Figure 7. Fixed-effect model forest plot of HR of OS in statistical analysis method subgroup analysis according to EGFR mutation status.** Solid diamond indicates the pooled HR of OS, Square indicates hazard ratio value of each study.  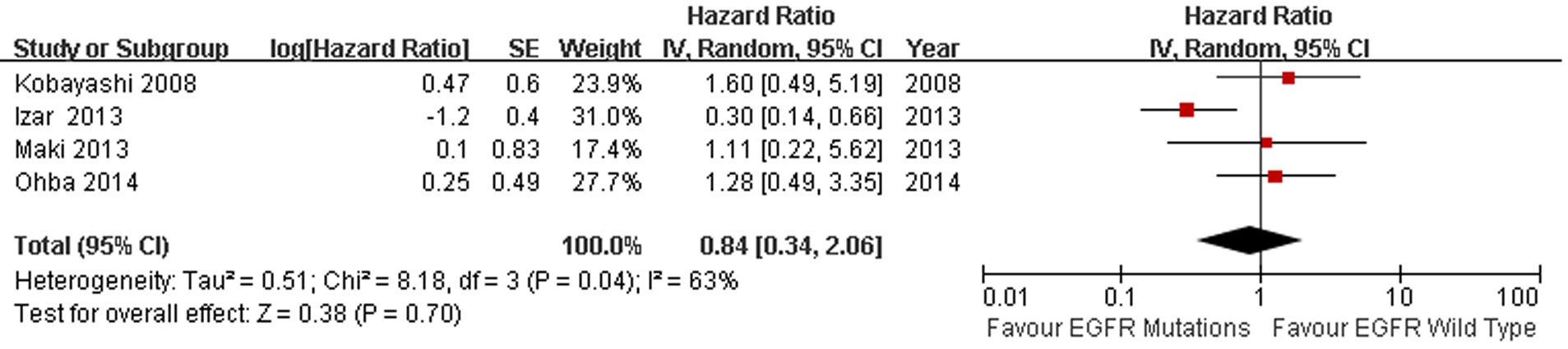  **Figure 8. Random-effect model forest plot of HR of OS in pathologic stage subgroup analysis according to EGFR mutation status.** Solid diamond indicates the pooled HR of OS, Square indicates hazard ratio value of each study. | Results |
| Synthesis of results | 21 | **Disease free survival:** HR 0.96, 95%CI [0.79-1.16] *P*=0.65. Figure 3. No heterogeneity was found (Chi^2^=13.15, *P*=0.16, I^2^=32%).  **Overall Survival**: HR 0.86, 95%CI [0.72-1.04] *P*=0.12. Figure 6. No Heterogeneity was found (Chi^2^=13.59, *P*=0.56, I^2^= 0%). | Results |
| Risk of bias across studies | 22 | Publication bias was not found according to funnel plots and Begg’s test by Stata 11.0. | Results |
| Additional analysis | 23 | **Disease free survival sensitivity analysis:** 11 studies compared the DFS between EGFR mutations and wildtype groups. Significant heterogeneity was detected among the studies (I^2^=62%, *P*=0.003). Heterogeneity did not change excluding any of studies but one in sensitivity analysis [27]. After excluding the study, there was no significant heterogeneity among the studies (I^2^=32%, *P*=0.16).  **Disease free survival subgroup analysis:** The subgroup analysis was performed according to the statistical analysis method in the survival outcomes and pathologic stage. There was no significant association between EGFR mutations and DFS. (multivariate analysis HR=0.89, 95%CI [0.71-1.12] *P*=0.32; univariate analysis HR=1.15, 95%CI [0.79-1.67] *P*=0.46 Figure 3). In the subgoup analysis according to pathologic stage, there was no significant heterogeneity among the 4 studies (I^2^=37%, *P*=0.19) .There was no association between EGFR mutations and DFS in stage I NSCLC patients. (HR=0.78, 95%CI [0.50-1.22] *P*=0.28 Figure 4).  **Overall Survival** **sensitivity analysis:** All of the studies compared the median survival time. We also conducted sensitivity analysis (Figure 6), though there was no significant heterogeneity was found in the studies (I^2^=35%, *P*=0.08). We found no heterogeneity among the studies when drop one study [27] (I^2^=0%, P=0.56).  **Disease free survival subgroup analysis:** The subgroup analysis was also performed according to the statistical analysis method in the survival outcomes and pathologic stage. The result of multivariate analysis did not show association between EGFR mutations and OS (HR=0.85, 95%CI [0.67-1.09] *P*=0.21, Figure 7); the result of univariate analysis also did not show the association (HR=0.88, 95%CI [0.67-1.15] *P*=0.34, Figure 7 ). There was no association between EGFR mutations and OS in stage I NSCLC subgroup. (HR=0.84, 95%CI [0.34-2.06] *P*=0.70 Figure 8). | Results |
| **DISCUSSION** | | |  |
| Summary of evidence | 24 | The systematic review with meta-analysis suggested EGFR mutations were not a prognostic factor in patients with surgically resected NSCLC. | Discussion |
| Limitations | 25 | Five studies were excluded for meta-analysis due to insufficient data to estimate the HR, Which may bring about the publication bias. The significant heterogeneity was found in the 11 studies eligible for meta-analysis of DFS. One important factor was frequency of follow-up and method of imaging. The time interval for surveillance was ranged from 3 months to 6 months after surgery. The shorter the follow-up time is, the earlier we can find the recurrence of the disease. Some studies used chest x-ray and abdomen ultrasound as method of imaging, while others used computed tomography scan of the chest and abdomen. There was also difference in using cranial computed tomography/magnetic resonance imaging. We couldn’t analysis by subgroup of frequency of follow-up and method of imaging, for many studies didn’t reporte enough detailed information. Many factors would influence the result of the meta-analysis: the baseline characteristics of the patients (included age, sex, pathologic stage, smoking history, pathological subtype). Smoking is the major co-founding factor for overall survival for lung cancer patient. 8 studies including 1937 patients on OS had multivariate analysis, and 2 of them including 977 patients analysed only after stage controlled [17, 23]. So the result of meta-analysis has to be treated cautiously. It is need to gather more studies to update the data in the future. Other biases found in the meta-analysis: We only used the full published papers; unpublished papers and meeting abstracts were excluded for not enough data; the method of extrapolation of HR maybe another potential source of bias. | Discussion |
| Conclusions | 26 | In conclusion, the systematic review with meta-analysis suggested EGFR mutations were not a prognostic factor in patients with surgically resected NSCLC. Well designed prospective study is needed to confirm the result. We can evaluate the prognostic value of EGFR mutation in more homogenous patients with stage I, especially stage IA, disease after surgery, for eliminating the disturb of any other treatment factors. | Discussion |
| **FUNDING** | | |  |
| Funding | 27 | Non | Non |

*From:*  Moher D, Liberati A, Tetzlaff J, Altman DG, The PRISMA Group (2009). Preferred Reporting Items for Systematic Reviews and Meta-Analyses: The PRISMA Statement. PLoS Med 6(6): e1000097. doi:10.1371/journal.pmed1000097

For more information, visit: **www.prisma-statement.org**.

Page 2 of 2
